# The Importance of Debiasing Social Media Data to Better Understand E-Cigarette-Related Attitudes and Behaviors

**DOI:** 10.2196/jmir.6185

**Published:** 2016-08-09

**Authors:** Jon-Patrick Allem, Emilio Ferrara

**Affiliations:** ^1^Keck School of MedicineDepartment of Preventive MedicineUniversity of Southern CaliforniaLos Angeles, CAUnited States; ^2^Information Sciences InstituteDepartment of Computer ScienceUniversity of Southern CaliforniaLos Angeles, CAUnited States

**Keywords:** Internet, surveillance, electronic cigarettes, Twitter, social media

In a recent issue of *JMIR,* Kim and colleagues described a framework for data collection, quality assessment, and reporting standards for social media data used in health research [1]. The authors’ framework was based on two principles: retrieval precision or “how much of retrieved data is relevant” and retrieval recall or “how much of the relevant data is retrieved.” With an in-depth knowledge of the subject matter under investigation, and refinement of the keywords to develop reliable search filters, the authors suggested that irrelevant content could be weeded out and high-quality data collection could be assured. Using the topic of electronic cigarettes (e-cigarettes), discussed on Twitter, as a case study to showcase their framework, the authors demonstrated how reporting standards could be made systematic and transparent. While the authors cogently argued for better reporting standards in social media data used in health research, and their principles regarding retrieval precision and retrieval recall were thoughtfully laid out, they overlooked the importance of identifying the sources of the content being captured during data collection. For example, Twitter has quickly become subject to third party manipulation where automated accounts are created by industry groups and private companies that aim to influence discussions and promote specific ideas or products [2]. This fact is absent from the framework of Kim and colleagues [1] and according to their principle of retrieval precision, researchers could classify tweets about e-cigarettes as high-quality data regardless of its origin.

Recent research has suggested that between 70% and 80% of tweets mentioning e-cigarettes stem from automated accounts [3]. Studies using tweets and that aimed at gaining insights to individual-level attitudes and behaviors are now faced with data with substantial bias and noise. Any results drawn upon this data and not preprocessed with de-noising techniques lose validity and significance. To ignore this bias in Twitter data would be akin to a public health researcher ignoring the bias from having a sample of participants, in a survey-based study on tobacco-related attitudes, where 700 of the 1000 participants happened to be gainfully employed by a tobacco company. The survey researcher would be forced to rethink their sampling frame, and the same dilemma applies to the social media researcher relying on Twitter as their data source. We propose herein that appropriate analyses be implemented to obtain valid data sets that remove sources of bias and noise before applying the framework of Kim and colleagues.

Twitter screen names responsible for each tweet collected in a data set should be obtained and each account’s recent history, interactions, and metadata should be analyzed to determine whether the account is a social bot, a computer algorithm designed to automatically produce content and engage with humans on Twitter [2]. These social bots are meant to appear to be individuals operating Twitter accounts that are complete with metadata (name, location, pithy quote) and a photo or an image. Tweets from these accounts pollute social and health research data sets and need to be identified and removed. Programs like “Bot Or Not?” [2] use a classification system that groups each Twitter account’s features into 6 main classes: Network (diffusion patterns), User (metadata), Friends (account’s contacts), Temporal (tweet rate), and Sentiment (content of message). This classification system ultimately generates a score that falls on a spectrum that can then be used to determine the likelihood of any one account being a social bot. If an account is identified as a social bot then that account and any tweets produced from that account should be removed from the dataset. This platform is freely available, easy to use, and has shown to be successful in reducing bias and noise in datasets from earlier studies led by computer scientists [2].

Using Twitter to examine e-cigarette-related discussion is a novel approach; however, the signal-to-noise ratio has become increasingly low [3]. In other words, the ratio of information representative of individuals’ perceptions, sentiments, and behavior is low as compared with the content from social bots. Prior studies have attempted to increase the signal-to-noise ratio by employing crude techniques (eg, removing any tweet that is accompanied by a URL [4]. However, this approach and other blunt approaches (eg, methods solely relying on community detection or methods solely relying on innocent by association paradigms—an account interacting with a human user is considered human) result in misclassification (eg, the removal of a valid tweet from the data set simply because it was accompanied by a URL or keeping an invalid tweet because a human interacted with the account it originated from) [5]. The debiasing techniques available to social media researchers proposed herein can be used to overcome earlier limitations.

Social bots are only one source of bias in studies of Twitter posts. For example, the population of Twitter users over represents young people and ethnic minority groups, when compared to the general population in the United States. This source of bias cannot be easily resolved by machine algorithms and correcting such biases should be a focus of future research. The use of social bots are not confined to discussions of e-cigarettes but have been found to infiltrate political discourse, manipulate the stock market, acquire personal information, and disseminate misinformation [5]. “Bot or Not?” is not a perfect system for bot detection, however, it scores a detection accuracy above 95% suggesting biases from inappropriate removal of legitimate accounts is minimal especially when compared with earlier approaches [5]. Researchers need to take advantage of the resources designed to reliably identify and remove third party accounts responsible for the noise in social media data. Once debiasing techniques have been exploited, frameworks for data collection, quality assessment, and reporting standards for social media data used in health research should be employed.
